# Implementing psychological interventions delivered by respiratory professionals for people with COPD. A stakeholder interview study

**DOI:** 10.1038/s41533-023-00353-8

**Published:** 2023-10-25

**Authors:** V. Wileman, V. Rowland, M. Kelly, L. Steed, R. Sohanpal, H. Pinnock, S. J. C. Taylor

**Affiliations:** 1 Wolfson Institute of Population Health, Barts and The London School of Medicine and Dentistry, Queen Mary University of London, 58 Turner Street, London, E1 2AB UK; 2 Health Psychology, School of Mental Health and Psychological Sciences, Institute of Psychiatry, Psychology and Neuroscience (IoPPN), King’s College London, 5th Floor Bermondsey Wing, Guy’s Campus, London Bridge, London, SE1 9RT UK; 3 School of Population Health and Environmental Sciences, King’s College London, Addison House, Guy’s Campus, London Bridge, London, SE1 9RT UK; 4 Allergy and Respiratory Research Group, Usher Institute, Doorway 3, Medical School, Teviot Place, Edinburgh, EH8 9AG UK

**Keywords:** Health services, Patient education, Chronic obstructive pulmonary disease

## Abstract

Implementing psychological interventions in healthcare services requires an understanding of the organisational context. We conducted an interview study with UK National Health Service stakeholders to understand the barriers and facilitators for implementing psychological interventions for people with chronic obstructive pulmonary disorder (COPD). We used TANDEM as an exemplar intervention; a psychological intervention recently evaluated in a randomised controlled trial. Twenty participants providing care and/or services to people with COPD were purposively sampled from NHS primary/secondary care, and commissioning organisations. Participants were recruited via professional networks and referrals. Verbatim transcripts of semi-structured interviews were analysed using thematic analysis. Four themes were identified: (1) Living with COPD and emotional distress affects engagement with physical and psychological services; (2) Resource limitations affects service provision in COPD; (3) Provision of integrated care is important for patient well-being; and (4) Healthcare communication can be an enabler or a barrier to patient engagement. People need support with physical and psychological symptoms inherent with COPD and healthcare should be provided holistically. Respiratory healthcare professionals are considered able to provide psychologically informed approaches, but resources must be available for training, staff supervision and service integration. Communication between professionals is vital for clear understanding of an intervention’s aims and content, to facilitate referrals and uptake. There was widespread commitment to integrating psychological and physical care, and support of respiratory healthcare professionals’ role in delivering psychological interventions but significant barriers to implementation due to concerns around resources and cost efficiency. The current study informs future intervention development and implementation.

## Introduction

For people living with chronic obstructive pulmonary disease (COPD), anxiety and depression are common comorbidities^1,2^. This is similar to other long-term physical conditions where people are two to three times more likely to experience mental health problems than those without a long-term condition^3^, yet people often face several barriers to accessing psychological support with services often separate to physical healthcare^4^. Integrated care refers to the concept of coordinated, patient-centred healthcare^5^ and integrated care systems outline the structure of collaborating services to deliver this^6^. For example, specialist pathways for people with long-term conditions (LTCs) are now embedded within the Improving Access to Psychological Therapies (IAPT) aiming to provide a harmonised care approach^7^. However, barriers to engaging with IAPT services are apparent for people with long-term physical conditions, including patient mobility limitations, unexpected illness exacerbations and accommodating physical healthcare appointments^8^. IAPT attendance for people with COPD has been reported as disproportionately lower compared with other long-term conditions and with poorer outcomes^9^.

There is a recognition overall that people should not be prevented from accessing physical healthcare services due to psychological challenges, and that psychological support should be incorporated into physical healthcare^10^. Increasingly across LTCs, physical healthcare professionals, including nurses and physiotherapists, are being encouraged to incorporate psychological approaches in their treatment provision^11–13^ and this has also been evidenced in COPD where specially trained and clinically supervised respiratory nurses have acquired the necessary skills to deliver CBT-informed treatment^14^. Their established credibility and trust with patients make physical healthcare professionals well placed to broaden their scope of treatment and some people with LTCs might prefer this approach to a separate referral to psychological services^15^. However, not all physical healthcare professionals feel able to provide psychologically informed approaches; others refer to the lack of skills, training and support required, and are concerned about limited resources to extend their treatment scope^16^. Therefore, integrating innovative models of care into existing healthcare systems needs to be considered from all perspectives before effective implementation can be achieved.

Successful implementation of healthcare research into practice requires that new practices and interventions be accepted and integrated by key stakeholders operating within or supporting healthcare service provision^17^. This paper reports a qualitative research study with respiratory health stakeholders to investigate the facilitators and obstacles influencing the implementation of an exemplar psychological intervention. TANDEM (Tailored intervention for ANxiety and DEpression Management in COPD), a self-management intervention based on a cognitive behavioural approach (CBA), aims to help improve anxiety and/or depression and encourage Pulmonary Rehabilitation (PR) uptake/completion. PR is considered part of gold standard treatment for COPD^18^ and there is increasing evidence that it may reduce the common co-morbidities of anxiety and depression^10^; however, PR uptake and completion levels, in the UK and worldwide, are poor. Approximately a third of patients referred to PR do not attend^19^, which is often explained by practical, organisational and communication barriers^20–23^ but for some people, co-morbid anxiety and depression might also prevent them from accessing and engaging with PR services^23,24^. Cognitive behaviour therapy (CBT) is a recommended treatment for supporting people with anxiety and depression^25^. In TANDEM (which also focusses on increased physical activity, reduced social isolation, and preparation for PR), understanding how mood, thoughts, symptoms, and behaviours relate to living with COPD is integral to all treatment sessions. By promoting techniques to reduce unhelpful thoughts and behaviour as well as managing physical symptoms, overall, the intervention aims to reduce anxiety and depression. Respiratory health professionals (nurses, physiotherapists, occupational therapists) were trained to deliver the intervention (40–60 min) face-to-face in the participant’s home, or NHS venue of their choice, weekly for 6–8 weeks, tailored to the presence and severity of the participant’s symptoms and need^26^.

TANDEM was recently evaluated in a pragmatic multi-site randomised controlled trial (RCT)^27,28^. This psychological intervention was delivered with high fidelity by the respiratory health professionals (Facilitators) trained to deliver the intervention and well received by participants, but outcome measures did not differ between groups at 6- and 12-month follow-up and the intervention was not found to be cost effective^29^. As part of the TANDEM process evaluation, we conducted a qualitative research study with respiratory health organisational stakeholders (COPD service commissioners, primary and secondary care COPD healthcare professionals, and psychologists), to reflect on how this intervention might complement their existing service provision. We wished to understand the resources and partnerships across different healthcare services, how psychological care is provided through existing services, and to explore any barriers and facilitators to implementation. Data collection and analysis for the current study were completed prior to trial completion; we report the findings unaltered and to inform future intervention development and implementation of comparable psychological interventions for people with COPD.

## Results

Of the 38 potential participants who were invited, 20 consented to participate. Reasons for non-participation included: no longer working in a relevant field, recommendation of colleague considered a more appropriate participant, inability to arrange interview dates and unknown (lack of response). Participants’ median duration of working within healthcare was 13 years (range: 2–30 years) and currently worked across different NHS settings (Table 1) in: London and Home Counties (*n* = 8), Midlands (*n* = 2), North-East (*n* = 2), North-West (*n* = 3), South-East (*n* = 4) and Wales (*n* = 1).

**Table 1:** Participants’ healthcare setting and roles.

Participants’ healthcare setting and roles (*n*)
Primary care
General Practitioner (2)
Respiratory Specialist Nurse (1)
General Practitioner & CCG Commissioner (2)
Secondary care
Respiratory Consultant (1)
Respiratory Consultant & CCG Commissioner (1)
Respiratory Physiotherapist (1)
Physiotherapy Lead for Community Respiratory Team (1)
Respiratory Consultant (1)
Liaison Psychiatrist (2)
Respiratory Nurse Specialist (1)
Pulmonary Rehabilitation Clinical Lead/Physiotherapist (1)
Respiratory Specialist Nurse (1)
IAPT/Primary Care Mental Health
Clinical Psychologist/High Intensity Therapist (1)
CBT Therapist (1)
Clinical Health Psychologist/IAPT Clinical Lead (1)
CBT therapist/IAPT Team Lead (1)
Clinical Psychologist/LTC Lead (1)

We report our findings under four distinct but related themes (Table 2). Within each theme, sub-themes were identified which explain the findings in more detail. The first theme “Living with COPD and emotional distress affects engagement with physical and psychological services” highlights the perceived complexity of living with COPD and how people need support with both physical and psychological symptoms across healthcare services. The second theme “Resource limitations affects service provision in COPD” underlines the challenges and considerations necessary for service provision such as, accessibility, funding and how services are delivered. The third theme “Provision of integrated care is important for patient well-being” describes the need for holistic services for COPD patients to ensure the most effective care. The final theme, “Healthcare communication can be an enabler or a barrier to patient engagement” highlights stakeholders’ perspectives that the communication about healthcare services (treatments) is a critical part of effective provision. This incorporates communication with those using the services but also within and between professional groups. Key enablers and barriers to psychological interventions like TANDEM are summarised in Table 3.

**Table 2:** Summary of themes.

Themes	Sub-themes
Living with COPD and emotional distress affects engagement with physical and psychological services	
Resources limitations affects service provision in COPD	Resources Accessibility Cost-efficiency
Provision of integrated care is important for patient well-being	
Healthcare communication can be an enabler or a barrier to patient engagement	Importance of effective patient communication about psychological interventions Communication between professionals

**Table 3:** Facilitators and Barriers to successful implementation of psychological interventions in COPD care.

Facilitators	Barriers
Integrated care aims to reduce the number of different services patients attend. Stakeholders supported combining traditionally separate treatments delivered by one healthcare professional.	Psychological interventions delivered by respiratory healthcare professionals might not be appropriate for all. People with significant psychological needs should be referred to mental health services.
Many people with COPD experience anxiety and depression; physical and psychological symptoms interact, and the person should be treated as a whole. Stakeholders considered that existing COPD service provision is often inadequate for this purpose and needs improvement.	Psychological interventions cannot simply be added to an existing service structure. Services, at a strategic level, need re-designing to incorporate this approach, linking primary and secondary care providers.
Stakeholders recognised that the benefits of pulmonary rehabilitation cannot be realised if people do not attend due to physical and psychological barriers. Treating people at home helps to remove these barriers.	Stakeholders are concerned about the perceived cost (and time) of home visits and considered that they might not be viable in geographically dispersed regions.
Additional benefits of home treatment were identified included how this might enable services to understand wider issues such as social well-being of the patients (e.g. housing, finance, family issues), which can also affect PR uptake.	Resources are a key concern; interventions must be able to demonstrate reductions in healthcare utilisation and cost-effectiveness. Doubts were expressed whether any intervention could reduce hospital admissions in a clinical population with the complexity of COPD.
Delivering psychological interventions could provide key skills to respiratory healthcare professionals and, over time, could become the norm.	Not all respiratory healthcare professionals will be able or willing to deliver such interventions and implementation must identify those who are best suited to the treatment approach. Equally, the provision of supervision, typical within a psychological service, must be addressed.
Additional benefits identified included being able to support other healthcare services, reducing the pressure of pulmonary rehabilitation and IAPT.	Referrers and providers must explain services effectively to improve uptake. COPD physical symptoms are real and worrying; offering a psychologically informed treatment needs to be carefully explained.

### Living with COPD and emotional distress affects engagement with physical and psychological services

Breathlessness, the loss of previous activity levels and social isolation were recognised challenges for people with COPD; they were also perceived as experiencing complex co-morbidities including other physical long-term conditions (e.g., cardiovascular disease and lung cancer) but also significant emotional distress including depression and anxiety.

*“There will be a cohort of the ones who are really more severe and have very high anxiety and or depression levels who really have a very poor quality of life and find it very difficult to engage in things”* Participant 2, Respiratory Consultant

Stakeholders recognised how emotional distress had an impact on people accessing services/treatment, particularly where people are anxious about leaving their home or attending group sessions, or when their depression symptoms affect their motivation and ability to participate.

*“You’ve got to get into the service, often you have to be fully engaged in the service before you have access to psychological interventions. And actually, what we’re trying to do often with rehab is to get someone to engage with the service in the first place.”* Participant 3, Respiratory Consultant & CCG Commissioner

It was also understood that many people with COPD are managing other difficult issues including “deprivation”, “trauma”, and “messy lives” and might struggle to engage with a psychological intervention. Additionally, it was noted that some people resist encouragement to talk about their mood:

*“…patients…really low in mood …I’ve reassured them and said…’Would you like to get some form of help with this?’ And they just go ‘No, I don’t want to talk about it to anyone. No.”* Participant 4, Respiratory Physiotherapist

*“They do need a different approach and that’s what I mean by what we offer at the moment, it’s very good for the mainstream, but we haven’t got much for those difficult to deal with patients. And usually, the reason is it’s…whatever it is, even if it’s pain or mobility, it’s usually steeped in their mental health, anxiety, depression, that’s fairly entrenched”* Participant 14, PR Clinical Lead/Physiotherapist

### Resource limitations affects service provision in COPD

As for many LTCs, the provision of healthcare services for people with COPD was described as multi-faceted, reflecting strategic, organisational and resource issues that affected the design and delivery of services.

#### Resources

Stakeholders described how resources were likely to be an obstacle to implementation and reflected particularly on their time restrictions, indicating the perspective that psychological interventions are seen as an additional service to usual clinical care. With limited resources in primary and secondary care, stakeholders were nervous about their capacity to integrate psychological interventions in their existing practice:

*“I think with the workload that it would bring; I think it would be too much for one person with having other duties as well…unless one individual was specifically employed to do this….and nothing else…”* Participant 4, Respiratory Physiotherapist

Delivering psychological interventions with less familiar skills and practices requires training, supervision, and rehearsal until they become more experienced and routine. Existing service structures were identified as difficult to change, particularly when working under pressure, which potentially inhibited putting new skills in to practice:

*“…this stuff takes practice…and you have to keep repeating the things that you learnt in order make them your default setting…when people go back into their working environments, there’s so much pressure to behave the way they used to…my sense is the desire is there, but the environment doesn’t allow you.”* Participant 11, General Practitioner & CCG Commissioner

However, optimism about the future was evident, recognising how such interventions would become a more familiar and standard approach and could be embedded into usual care and complement existing physical health services.

*“Some people, if they were more negative might say, ‘What? Not more things to deliver’. But you’d have to make sure that there was enough staff to provide it, because I think it [psychological care] does… it is an extra… But ultimately how that will then feed down, the trickle effect into the wider team, everybody will start to be coming from a more CBT approach.”* Participant 14, PR Clinical Lead/Physiotherapist

Resources were also considered in terms of priorities and tangible benefits, with the recognition that patient benefit alone is not enough to ensure commissioning of new service approaches. Some stakeholders made suggestions for alternative ways of delivering psychological interventions that might reduce costs.

*“I mean for the patient, it’s about whether they feel better, whether it improves their quality of life. But unfortunately, that’s not enough to create change. …I guess face-to-face CBT at home is probably quite expensive, was one thought…if you could do it by phone it would be much more time efficient, wouldn’t it? And therefore, likely to be cost efficient.”* Participant 2, Respiratory Consultant

As IAPT services increased their provision in LTCs, psychological support has become more available for people with COPD. Yet some IAPT services are also under resourced, and resource-driven online services might not be appropriate in this context.

*“IAPT is overstretched and in order to meet targets…and also from some very genuine thoughtful reasons there’s a focus on online stuff and groups. However, lots of these populations, they’re older or they’re from minority groups that might mean that actually a group is quite stigmatising, quite threatening. Or they don’t have access to IT, or they just don’t want that sort of intervention. And a lot of them are also housebound or very physically unwell. So, engaging with that sort of service is just by definition really difficult….”* Participant 8, Liaison Psychiatrist

IAPT professionals recognised psychological interventions like TANDEM, delivered by respiratory healthcare professionals, as a potential complementary service, particularly to meet the needs of those patients who could not engage with current IAPT provision. However, pursuing additional psychological provision outside of IAPT services was also seen as an obstacle in securing commissioning within respiratory services.

*“…you can anticipate in a cost pressured environment; the question will be ‘Why can’t we do this via IAPT’”?* Participant 6, Respiratory Consultant

#### Accessibility

Considering the provision of psychological interventions like TANDEM in usual care, stakeholders described COPD services in the context of geographical variations and how services could be most effectively and efficiently resourced as well as considering patient accessibility barriers. For some regions, where patients’ locations are widely dispersed, the potential for healthcare professionals visiting patient homes was more restricted and unlikely to be resourced. The creation of COPD healthcare ‘hubs’ was discussed by different stakeholders, with aligned services in one community location, providing a holistic service:

*“It’s good for things to be in the community, we’re trying to achieve a middle ground between the patients coming a little way and the clinician coming some way…”* Participant 3, Respiratory Consultant & CCG Commissioner

Despite the geographical barriers, the notion of visiting people at home was supported by stakeholders who recognised the physical and psychological challenges that many people with COPD experience and also appreciated the benefits that extend beyond the core treatment task:

*“…. whereas if you’re able to do home visits that will open up a lot of doors for patients who can’t always get out to many appointments and that. And who struggle to leave the house because of anxiety or depression or any form of psychological problem. That would really help them to build up their confidence. And then make them feel able to then go out and come to pulmonary rehab,”* Participant 4, Respiratory Physiotherapist

Moreover, home visits could offer the opportunity for services to understand other surrounding issues such as the social well-being of the patients, which can affect PR uptake.

*“…. when people go out to the house you often get all the added benefit of that social interaction…. this person’s really struggling at home, we need to get the social work involved and stuff.”* Participant 6, Respiratory Consultant

#### Cost-efficiency

Limited resources allocated to the management of respiratory disease generally was identified as a barrier to implementation. There was a shared expectation that to be considered for commissioning, an intervention must be able to demonstrate significant reductions in healthcare utilisation in addition to improving patient outcomes:

*“The key metrics are going to be, does it reduce hospital admissions…and does it reduce GP visits,”* Participant 2, Respiratory Consultant

Concern was expressed, however, about whether any intervention could reduce hospital admissions in a clinical population with the complexity of COPD, where people are often living with multiple conditions and can be very unwell:

*“Will it impact on admissions?…very difficult to do…. they’re very, very difficult people to keep out of hospital…that’s the problem”* Participant 10, General Practitioner & CCG Commissioner

Stakeholders considered the provision of core COPD services in the context of the NHS long-term plan and described the focus on improved diagnostics (spirometry) and the expansion of PR services, which are considered to be cost-effective, although this is not realised if patients are not able to attend and engage. Priorities and measurable outcomes continued to be cost driven.

*“…we know that pulmonary rehab is probably the single most effective treatment for breathlessness and COPD… It’s incredibly cost effective…But the challenges in rehab delivery are getting people to the programme and getting them through to complete…”* Participant 3, Respiratory Consultant & CCG Commissioner

Stakeholders recognised that commissioning funds were needed to support people with COPD experiencing anxiety and depression. Stakeholders referred to inconsistent priorities in commissioning and, for some, doubt existed as to whether respiratory was an important funding area.

*“I don’t think respiratory is very high on the Government’s agenda. I think historically respiratory is poorly funded when you compare it to other diseases such as heart disease and things.”* Participant 9, Respiratory Specialist Nurse

### Provision of integrated care is important for patient well-being

Stakeholders agreed about the importance of integrated care. They recognised delays in accessing psychological services, and that patients were not always able or willing to engage with additional services, instead preferring the established relationship with their respiratory healthcare professional. Some psychological services were not considered specialist enough for respiratory patients, with sufficient understanding of the symptom burden of COPD and the physical health care needs and flexibility often required by people with COPD.

*“…IAPT still work with mild to moderate presentations, which because of the nature of some long-term conditions they don’t always fit nicely into that box to put it…. I feel that we’re a little bit limited as practitioners and as a service when certainly my experience, what I feel like, within IAPT, when we work with this cohort [COPD] of clients.”* Participant 15, CBT Therapist

A multidisciplinary team model was considered an effective approach and stakeholders from psychology services were concerned that if the treatment was not delivered as outlined in the evidence base, that it might be ineffective, and patients might conclude that such approaches won’t work for them.

*“…there’s a danger in having too light a psychological treatment. That actually, you don’t provide what the evidence base says you should do. And that, in itself, might put people off psychological work because they may say ‘that doesn’t work’ or ‘it doesn’t work for me’ or whatever…”* Participant 16, Clinical Health Psychologist/IAPT Clinical Lead

Stakeholders were positive about respiratory healthcare professionals providing psychological support to their patients. Many respiratory nurses and physiotherapists were perceived to have key psychological competencies in their clinical practice, although it was suggested that not all physical healthcare professionals would be prepared to offer psychological support.

*“…they’re actually some of the best placed people to do it, particularly when the mood issue is very much related to the chronic condition that exists, because their knowledge of that condition and the volume of people they see, what’s normal for the illness, what isn’t, how have other people managed it, etc.”* Participant 12, General Practitioner

*“…individuals within services who are willing to explore new techniques…. a handful of the people who could possibly offer it. Because other staff members felt that it was, I don’t know, wasn’t for them, and didn’t feel like they could deliver it…”* Participant 9, Respiratory Specialist Nurse

Queries were raised about how physical healthcare professionals would be supervised and supported as is the norm in psychological services.

*“My only concern would be ‘Is that an appropriate and sufficient level of support?’… supervision, so that there’s somewhere that contains the worries and anxieties which can come about from managing a very, very complex caseload…”*. Participant 7, Liaison Psychiatrist

Stakeholders supported TANDEM’s aim, objectives, and approach and identified additional factors on which comparable psychological interventions could have a positive impact, such as social well-being. The approach was deemed to be holistic, integrative, empowering, supportive, and complementary of current services.

*“So, my instinctive response to it [TANDEM] is, I think it’s superb, I think these are core skills, these are hugely common comorbidities. I think the integrated respiratory team people should have these skills. I think having to put everything into mental health is the wrong way to look at it….”* Participant 8, Liaison Psychiatrist

### Healthcare communication can be an enabler or a barrier to patient engagement

Communication about healthcare services with both patients and healthcare professionals is a critical part of effective provision—to ensure that patients fully understand what treatments involve and how they can help them manage their COPD, and to address any concerns they might have. When this communication is accurate and clearly understood, stakeholders recognise this as an important step towards patient engagement.

#### Importance of effective patient communication about psychological interventions

Talking with patients about how thoughts and feelings might relate to their breathlessness has to be carefully explained to validate the symptom burden that many experience. Stakeholders described how patients can respond negatively if they suspect it is being suggested that their breathless symptoms are not real physical symptoms:

*“…. you imagine somebody coming and saying ‘Doc, I’ve got really bad COPD, there’s my breathing tests, they’re terrible’. And me saying ‘You know what? You need to go and see somebody about how you feel stressed or not….’ You’re dead breathless, therefore you must go and see a psychologist. Well, that means…’are you denying I’ve got a physical disease?’ ‘Do you think I’m making up all this breathlessness?’“* Participant 6, Respiratory Consultant

Many stakeholders compared the challenge of effectively communicating about psychological interventions with the existing problem of the misunderstanding that surrounds PR and how improving this is also important for uptake to healthcare services and overall patient well-being. Stakeholders considered that referrers do not always explain PR to patients as well as they could, and as such, patients are less likely to engage, although referrers felt the terminology (“pulmonary rehabilitation”) was a barrier in itself to effective communication and requires better explanation.

*“So, the whole concept of PR is utterly alien to a patient,”* Participant 3, Respiratory Consultant & CCG Commissioner

*“I wish we could rebrand it or rename it. I think ‘rehabilitation’ or ‘going to rehab’ is just…it sounds like drink or drugs or…”* Participant 19, Respiratory Specialist Nurse

#### Communication between healthcare professionals

Stakeholders acknowledged that often the communication between healthcare professionals about some services, is also ineffective, which affects uptake and ultimately might affect future commissioning. Recognising the importance of understanding and communicating about services between providers and referrers is an important factor for implementing new psychological interventions in usual care:

*“I think healthcare professionals still don’t understand the significance of it,”* Participant 9, Respiratory Specialist Nurse

*“And so, there’s that issue of convincing commissioners, here’s the evidence, and that’s important to you now, and then there’s the whole thing is we’ve commissioned the service, and nobody turned up. And so, the example that was pulmonary rehab programmes have been commissioned in my area, and they haven’t turned up because the communication hasn’t been very good, through stakeholders like GPs.”* Participant 6, Respiratory Consultant

## Discussion

The participants in our study, represented a variety of physical and psychological health service providers, offering the breadth of perspectives we sought to explore. We found a shared commitment to improving services for both the physical and psychological impact of COPD, with support for integrated psychological approaches such as TANDEM, which are delivered by respiratory professionals. However, organisational barriers were highlighted with concerns about the provision of psychological treatment and the skills, structure and resources required.

TANDEM is an example of a holistic intervention approach which recognises the impact of physical symptoms on psychological symptoms and vice versa, and advocates how respiratory professionals can provide both as part of routine care, which has been evaluated in a previous COPD trial with effective outcomes^14^. Stakeholders recognised inadequacies with existing psychological services for COPD and were mostly confident that with appropriate training and supervision support, respiratory healthcare professionals could incorporate psychological approaches. However, psychology services highlighted concerns that if the psychological treatment provided in this context was insufficient, it might leave people feeling any psychological support wouldn’t help them. The exemplar intervention (TANDEM) was designed to be similar to low intensity IAPT so arguably this potential problem is not confined to healthcare professionals providing such support. However, in IAPT, people can be referred to higher intensity services and it is important that referral processes are clearly defined for people who require more specialist psychological support. This stratified approach is also seen in other physical healthcare sectors such as cancer services^30^ and kidney disease^31^. Findings from this study also support previously reported barriers for services like IAPT providing psychological support for people with LTCs^8,9^. IAPT professionals acknowledged that their services are not always sufficiently flexible for some people with LTCs, and research has indicated that IAPT provision within physical care services might be preferable to patients^8^, for example, in an integrated treatment centre approach.

For many COPD patients unable to leave the home easily to access care services, an alternative approach such as home visits, may be needed. Our study found that home visits were valued, particularly where other problems could be identified, such as social care issues. Providing COPD services at home has been considered previously^22^, and whilst this has been explored as a vehicle for psychological interventions like TANDEM, there has also been increasing interest in home-based PR itself to overcome barriers for people unable to attend elsewhere^32,33^. However, financial constraints were a concern, particularly resourcing teams to support a patient population which was geographically dispersed. Stakeholders asked whether psychological interventions like TANDEM could be delivered remotely (by phone/remote consulting) to help with costs. Remote healthcare has been widely used during the Covid pandemic and remote services, such as smoking cessation support and self-management interventions have been established^34^. However, TANDEM trial Facilitators spoke of the “complexity of the therapeutic process” (paper under review) and arguably, remote contact might hinder this process.

Stakeholders recognised that not all respiratory healthcare professionals would be able or willing to deliver psychological support as it was considered an additional and specialist skill. The TANDEM research group recognised the importance of this issue when recruiting Facilitators for the trial, and evaluated whether applicants had an understanding and enthusiasm for working with a psychologically informed approach. Applying the same rigour in identifying respiratory healthcare professionals whose treatment approach fitted with the model, and assessing competence, would be essential for successful implementation of psychological interventions in routine care.

Healthcare professionals’ understanding and subsequent ability to communicate PR to patients has previously been identified as a barrier to services uptake^21^ and this study supports these findings. Acknowledging this existing communication problem and pursuing improvement in terminology and explanations is important as further communication challenges are anticipated in relation to talking with people about how mood might relate and respond to their COPD symptoms. It is imperative to agree a meaningful and acceptable shared language with all stakeholders and people with COPD, to limit such barriers to both PR and psychological support.

In common with other recent research studies^35^, a key concern by many stakeholders, was the lack of resources available to incorporate what might initially be viewed as an extra service, although it was recognised that the approach could, over time, be embedded and accepted in usual practice. In a resource-constrained NHS, competing demands for services will always be an issue and a challenging decision for commissioners. Respiratory medicine was considered by some stakeholders to lack the attention and resources it needed compared with other conditions.

The main strength of this study is taking a contextual perspective with key stakeholders responsible for implementing services, involving representatives from healthcare services that provide care to people with COPD; to ask them how this treatment could complement and enhance their existing provision or whether they could advise us of anticipated barriers that would need to be addressed. A particular strength is the inclusion of many stakeholders from psychology services within secondary care COPD services as well as separate IAPT organisations, to learn about their experiences in supporting people with COPD.

In our attempts to invite stakeholders from a variety of services and from different geographical regions, we recognise we have relatively few representatives from each service type. We also appreciate that our recruitment methods, especially professional colleague referrals, are likely to have attracted participants particularly interested in psychological support in COPD and therefore they may have expressed more enthusiasm towards the aims of our exemplar intervention (TANDEM) than others may hold. Additionally, as our participants were based in UK NHS services, these findings may not be directly transferable to different healthcare systems and models internationally. However, the issues surrounding COPD and anxiety/depression are recognised globally as is the challenge of delivering holistic accessible care.

Authors VW and VR, who conducted interviews, analysed data, and prepared this manuscript were employed on the TANDEM trial and understand the potential bias of this position. To counteract this bias, we actively screened the data for content describing potential barriers to implementation. We also made sure in our sampling strategy that that we had representatives from psychological services for people with COPD, to reflect their views on non-psychologists providing this approach. Further, both were independent of intervention development, VW was independent of trial management, and we conducted the study analysis and write-up, prior to trial outcomes announcement with no changes to the reported findings.

Stakeholders were positive about training respiratory healthcare professionals in psychological aspects of care whilst recognising not all of them will be suitable to deliver it, and some patients with greater psychological needs will require more specialist mental health support. This study suggests that respiratory healthcare professionals and psychologists may therefore both have a role in providing levels of psychological care appropriate to patients’ requirements. Working within a resourced constrained health service and considering COPD patients’ varying physical and psychological needs, perhaps the model of “COPD treatment hubs” where respiratory healthcare professionals and psychologists specialising in COPD provide integrated services side-by-side should be explored further. Previous literature has referred to a similar service arrangement and acknowledge the improved knowledge about the different aspects of COPD care that occurs between professionals in this environment^35^. Given the stakeholders perspectives on the need for improved communication between professionals about COPD services, this would potentially be an important benefit. Finally, further consideration should be given to embedding psychological approaches and skills in healthcare foundation training.

This study highlights how implementing interventions in COPD care is particularly complex compared with some other LTCs as many are burdened with multiple health conditions and experience barriers to accessing services. Facilitators for implementation included that stakeholders were supportive of improving psychological care for people with COPD and integrating it into current service provision. However, barriers were identified, particularly resources, that will need addressing to ensure the successful implementation of psychological interventions delivered by respiratory healthcare professionals, as well as a shared commitment to resourcing, embracing, and embedding new ways of working.

## Methods

### Study design and sample

A qualitative study was undertaken to explore the perspectives of stakeholders involved in the provision of respiratory care in England and Wales, regarding the prospective implementation of TANDEM in the NHS. We communicated and invited expressions of interest to participate in our research study via brief notices on relevant social media such as Twitter https://twitter.com/TANDEMcopd and related networks e.g. https://www.respiratoryfutures.org.uk/, www.chain.org and https://twitter.com/respfutures. Potential participants were also identified by contacts across professional networks. Guided by the resources available for the study, we planned to recruit a purposive sample size of up to 20 participants from NHS primary and secondary care settings, including: GP surgeries, clinical commissioning groups (CCGs) who plan and commission healthcare services, pulmonary rehabilitation and respiratory services, and mental health services (psychologists delivering IAPT services). Participants needed to be currently or recently (<12 months) providing care to people with COPD, referring to PR or commissioning PR services. Participants were not involved in delivering TANDEM.

### Data collection

We interviewed participants between November 2019 and January 2020 (RCT recruitment June 2018 to March 2020). Interviews were conducted by VW (Psychologist, PhD) and VR (Trainee Health Psychologist, MSc), both experienced in qualitative methods; neither were known to the participants previously. Data were collected via telephone interviews using a semi-structured topic guide (Supplementary File), developed, and tested in consultation with the TANDEM process evaluation team. The average interview length was 40 min (17–83 min).

### Data analysis

Audio recorded interview files were transcribed verbatim by external transcription services and checked by VW or VR for accuracy. Using NVivo software v12, the data were coded and analysed by VW and VR. We maintained reflexivity by recording our understanding about the data and judgements about the analysis process. We adopted a realist epistemological perspective which assumes a unidirectional link between participants’ experiences, meaning and responses as outlined by Braun and Clarke^29,36^. We applied an inductive method of analysis where themes were generated at a semantic level, describing and interpreting participants’ words at face value. We followed Braun and Clarke’s recommended coding reliability process, including familiarisation with the data, generating initial codes, searching, reviewing and finally, defining themes. The first round of coding consisted of independently coding two transcripts to identify content and generate initial themes. We compared themes, reviewed any inconsistencies, and developed a draft coding framework. We coded three further transcripts simultaneously to check for consistent interpretation and agreed a final coding framework. We were satisfied with the level of consistency and divided the remaining transcripts equally. Once coding was completed, we met regularly to discuss and structure themes for coherence. The salient data extracts were selected to include in the findings. Summaries were presented and shared with the TANDEM process evaluation team for discussion.

### Consent and ethical issues

The study was funded by NIHR, and ethical approval was granted by the London-Queen Square Research Ethics Committee, reference 17/LO/0095. Written informed consent was obtained from all participants. The anonymised transcripts were stored digitally on an encrypted USB and audio files were subsequently deleted from the encrypted recorders.

### Supplementary information

Supplementary files

nr-reporting-summary.pdf

## Data Availability

The Pragmatic Clinical Trials Unit (PCTU) at Queen Mary University of London has developed a data sharing policy to facilitate controlled access to data from PCTU studies. For all data sharing requests, please contact the PCTU and complete a Data sharing Request Form. For enquiries about data sharing: pctu-data-sharing@qmul.ac.uk.
