# Supplementary material for: Implementing psychological interventions delivered by respiratory professionals for people with COPD. A stakeholder interview study
Source: NPJ Prim Care Respir Med. 2023 Oct 25;33:35. doi: 10.1038/s41533-023-00353-8 (PMC10600190; doi:10.1038/s41533-023-00353-8)
Supplement: Supplementary file 1 — Supplementary files [file 41533_2023_353_MOESM1_ESM.pdf]

# Supplementary Notes

**Topic Guide A (Participants who provide PR services)**

1. Please can you explain your role and what services your organisation provides in relation to COPD/PR? Prompts: expertise, responsibilities, time in role.

2. What are the key issues you are facing in delivering and improving services/service uptake for COPD? What approaches have you previously applied to the improved management and/or quality of life of this patient group?

3. What are your views on using an alternative approach to address the psychological/emotional well-being of patients with COPD? Prompt for any previous knowledge/experience

4. Have you heard about the TANDEM research project? If yes, could you tell me about your understanding of the TANDEM intervention and aims/objectives?

   Provide a brief description of TANDEM see Page 5 – use/adapt as appropriate depending on response to item 4.

5. What are your initial thoughts on this approach (TANDEM)?

6. How might the aims and methods of TANDEM complement/differ from your current services?

7. Who would be involved in the implementation and on-going delivery of TANDEM in your organisation? Prompt: what would your role be, who would you work with to implement this?

8. What would need to be established in order for TANDEM to operate in your organisation?

9. How do you think your patients (if relevant) would respond to TANDEM? Prompt, would some patients be more suited to TANDEM than others?

10. What are your views on respiratory HCPs delivering a psychological approach with patients with COPD? Prompt – if mentions IAPT as an alternative service, ask about their experiences of referring to IAPT and observed outcomes. Prompt: If a service has psychology support already in situ, prompt response to envisage how this would complement existing support and to consider how it might operate in a service without it. Prompt - if response suggests nurses/physios might **not** be equipped to manage psychological intervention, prompt why, what would they need in order to feel equipped etc.

11. What do you feel would be the key facilitators and/or barriers to implementation? Prompts: are there organisation of practical issues that you think might be important?

12. What are your thoughts on this being commissioned in future? How likely? Prompt for reasons if not

13. How would the service be delivered in the future? Who, when, how organised? Prompt for any current appraisal methods for existing/new services.

**Topic Guide B (GPs / Practice Nurses)**

1. Do you refer people with COPD for pulmonary rehabilitation (PR)?
   If yes – how do you feel PR benefits your patients with COPD? If not, prompt for reasons e.g., other practitioners refer.

2. Are there any particular types/groups of patients with COPD that you refer to PR? Are there any particular patients with COPD you would not refer/ would be less likely to refer? Prompt patient characteristics that relate to referrals/outcomes from PR…pragmatic issues, psych, timing, access, waiting lists?

3. What are the key issues you are facing in supporting people with COPD including their referral to PR? Prompt: Psychological health, other co-morbidities

4. What approaches (if any) have you previously applied to the improved management of this patient group? Prompt, other support services (clinical and non-clinical). Prompt, what worked, what didn't and why?

5. Have you heard about the TANDEM research project? If yes, could you tell me about your understanding of the TANDEM intervention and aims/objectives?

   Provide a brief description of TANDEM see Page 5 – use/adapt as appropriate depending on response to item 5.

6. What are your initial thoughts on this approach (TANDEM)?

7. What are your views on respiratory nurses and physios delivering this sort of approach (with training) with patients with COPD? Prompt - participant might for example suggest IAPT as an alternative service, ask about their experiences of referring to IAPT and observed outcomes. If response suggests nurses/physios might not be equipped to manage psychological intervention, prompt why, what would they need in order to feel equipped etc

8. How do you think your patients with COPD (if relevant) might respond to an intervention like TANDEM? Prompt, do you envisage any barriers to participants engaging with this treatment?

9. How might the aims and methods of TANDEM complement/differ from the current services that are currently available for your patients with COPD?

10. What do you feel would be the key facilitators and barriers to implementation?

11. Do you think the service would be commissioned in future? Prompt for reasons if not

12. How would the service be delivered in the future? Who, when, how organised? Prompt for any current appraisal methods for existing/new services.

**Topic Guide C (Clinical Commissioning Groups)**

1. Can you tell me about your role and organisation? Prompt – explore pressures and targets – deliverables short and long term?

2. What are the key priorities for the CCG, now and over the next 5/10 years? Prompt for COPD relevant priorities. What do you have to deliver – how will it be measured? Prompts – financial pressures, political influences – other services demanding time and resources

3. What services are you currently commissioning related to COPD? Prompt for insight into LTCs and specifically COPD. What other services are you commissioning?

4. What are the main influencing factors on decision-making when commissioning services – patient outcomes, savings?

5. Thinking about integrated care (mind-body model / psychological and physical health), what does that look like from your perspective?

6. Who do you see delivering these services (Prompt - e.g., IAPT)?

7. Have you tried anything similar (integrated physical and mental health services) …what worked, what did not? Where (PCT/Secondary care)?

8. Have you heard about the TANDEM research project? If yes, could you tell me about your understanding of the TANDEM intervention and aims/objectives?

   Provide a brief description of TANDEM see Page 5 – use/adapt as appropriate depending on response to item 8.

   Explain full cost analysis will be an integral part of study outcomes and ask for any initial thoughts on this approach, explore any opportunities, ideas, barriers.

9. What do you think of this approach and how it might be delivered?

**Topic Guide D (Integrated mental and physical health services for long term conditions)**

1. Can you tell me about your role and organisation? Prompt for how long they have worked within this service and background/experience? Prompt about Supervision – how this might be provided in future.

2. What services are you delivering – where does this take place? How are people referred to you? Prompt for some detail e.g., how many people, how often, structure of treatment programmes etc

3. Where do you usually get your referrals?

4. Do you treat people with long-term conditions? Prompt for types of LTCs, experiences of what has worked and what has not worked.

5. Have you provided services to people with COPD? Prompt as above.

6. What are your eligibility criteria for someone with COPD to be seen?

7. Where is someone referred to if they do not meet your eligibility criteria?

   <u>IAPT SPECIFIC:</u>

8. What LTCs does your funding focus on? i.e., COPD, Diabetes etc

9. Do you feel you are adequately trained to work with people with LTCs and low-level mood problems?

10. What training have you been involved in for LTCs? (Prompt: length of training / delivered by?)

11. Have you heard about the TANDEM research project? (If relevant - Could you tell me about your understanding of the TANDEM intervention and aims/objectives?)

    Provide a brief description of TANDEM see Page 5 – use/adapt as appropriate depending on response to item 11.

12. What are your initial thoughts on this approach (TANDEM)? Prompt: perception about service delivery, how might it complement their service, are there any overlaps? Prompt about supervision, who might provide it, might IAPT have a role here?

13. What do you feel would will be the key challenges/barriers to delivering TANDEM?

14. How do you think TANDEM would be perceived by your team, if implemented? (Prompt: welcomed? Complement / compete with your service?)

**Brief description of the TANDEM intervention**

TANDEM is a tailored intervention for people diagnosed with moderate to very severe COPD experiencing mild/moderate anxiety and/or depression prior to attendance in pulmonary rehabilitation (PR). TANDEM treatment links two proven approaches: psychological (talking) treatment with physical (pulmonary rehabilitation) treatment. We believe addressing mood before starting PR may help people with COPD as many sufferers experience anxiety around the idea of exercise. Our new intervention involves up to eight face-to-face visits (home or GP practice) by a trained respiratory HCP to COPD patients. After this they will receive continued support by phone calls through PR with their

local service. The respiratory HCP will be trained to deliver a cognitive behavioural approach which will be tailored to the needs of patients.

Questions that we hope to answer about the new intervention are: Can it help manage anxiety and depression? Does it encourage sufferers to start (and finish) a PR course? Will it lead to reduced breathlessness, increased exercise capacity and wellbeing, reduced days spent in hospital? How much would it cost the NHS?

We are interested in learning from people who manage or refer people to PR how this new treatment is perceived, how it might fit with current services, any barriers that need to be considered – so that if it is found to be more effective than existing treatments, it can be successfully implemented into clinical practice.
