# Supplementary material for: Implementing psychological interventions delivered by respiratory professionals for people with COPD. A stakeholder interview study
Source: NPJ Prim Care Respir Med. 2023 Oct 25;33:35. doi: 10.1038/s41533-023-00353-8 (PMC10600190; doi:10.1038/s41533-023-00353-8)
Supplement: Supplementary file 2 — nr-reporting-summary.pdf [file 41533_2023_353_MOESM2_ESM.pdf]

nature portfolio

Corresponding author(s): Dr Vari Wileman NPJPCRM-01649R

Last updated by author(s): Sep 4, 2023

Reporting Summary

Nature Portfolio wishes to improve the reproducibility of the work that we publish. This form provides structure for consistency and transparency in reporting. For further information on Nature Portfolio policies, see our Editorial Policies and the Editorial Policy Checklist.

Statistics

For all statistical analyses, confirm that the following items are present in the figure legend, table legend, main text, or Methods section.

n/a	Confirmed	
☒	☐	The exact sample size (*n*) for each experimental group/condition, given as a discrete number and unit of measurement
☒	☐	A statement on whether measurements were taken from distinct samples or whether the same sample was measured repeatedly
☒	☐	The statistical test(s) used AND whether they are one- or two-sided<br>*Only common tests should be described solely by name; describe more complex techniques in the Methods section.*
☒	☐	A description of all covariates tested
☒	☐	A description of any assumptions or corrections, such as tests of normality and adjustment for multiple comparisons
☒	☐	A full description of the statistical parameters including central tendency (e.g. means) or other basic estimates (e.g. regression coefficient) AND variation (e.g. standard deviation) or associated estimates of uncertainty (e.g. confidence intervals)
☒	☐	For null hypothesis testing, the test statistic (e.g. *F*, *t*, *r*) with confidence intervals, effect sizes, degrees of freedom and *P* value noted<br>*Give P values as exact values whenever suitable.*
☒	☐	For Bayesian analysis, information on the choice of priors and Markov chain Monte Carlo settings
☒	☐	For hierarchical and complex designs, identification of the appropriate level for tests and full reporting of outcomes
☒	☐	Estimates of effect sizes (e.g. Cohen's *d*, Pearson's *r*), indicating how they were calculated

Our web collection on statistics for biologists contains articles on many of the points above.

Software and code

Policy information about availability of computer code

Data collection	n/a
Data analysis	n/a

For manuscripts utilizing custom algorithms or software that are central to the research but not yet described in published literature, software must be made available to editors and reviewers. We strongly encourage code deposition in a community repository (e.g. GitHub). See the Nature Portfolio guidelines for submitting code & software for further information.

Data

Policy information about availability of data

All manuscripts must include a data availability statement. This statement should provide the following information, where applicable:

- Accession codes, unique identifiers, or web links for publicly available datasets
- A description of any restrictions on data availability
- For clinical datasets or third party data, please ensure that the statement adheres to our policy

The data that support the findings of this study are available from the corresponding author upon reasonable request subject to appropriate data sharing permissions

Research involving human participants, their data, or biological material

Policy information about studies with human participants or human data. See also policy information about sex, gender (identity/presentation), and sexual orientation and race, ethnicity and racism.

Reporting on sex and gender	We have not collected these data
Reporting on race, ethnicity, or other socially relevant groupings	We have not collected these data
Population characteristics	See above
Recruitment	We communicated and invited expressions of interest to participate in our research study via brief notices on relevant social media such as Twitter https://twitter.com/TANDEMcopd and related networks e.g. https://www.respiratoryfutures.org.uk/, www.chain.org and https://twitter.com/respfutures. Potential participants were also identified by contacts across professional networks. Written informed consent was obtained from all participants.
Ethics oversight	London-Queen Square Research Ethics Committee, reference 17/LO/0095.

Note that full information on the approval of the study protocol must also be provided in the manuscript.

Field-specific reporting

Please select the one below that is the best fit for your research. If you are not sure, read the appropriate sections before making your selection.

☐ Life sciences ☒ Behavioural & social sciences ☐ Ecological, evolutionary & environmental sciences

For a reference copy of the document with all sections, see nature.com/documents/nr-reporting-summary-flat.pdf

Behavioural & social sciences study design

All studies must disclose on these points even when the disclosure is negative.

Study description	We conducted an interview study with UK National Health Service stakeholders to understand the barriers and facilitators for implementing psychological interventions for people with chronic obstructive pulmonary disorder (COPD).
Research sample	Participants providing care and/or services to people with COPD were purposively sampled from NHS primary and secondary care, and commissioning organisations
Sampling strategy	Purposive sampling
Data collection	Data were collected via telephone interviews using a semi-structured topic guide
Timing	November 2019 and January 2020
Data exclusions	No data exclusions
Non-participation	Of the 38 potential participants who were invited, 20 consented to participate. Reasons for non-participation included: no longer working in a relevant field, recommendation of colleague considered a more appropriate participant, inability to arrange interview dates and unknown (lack of response).
Randomization	n/a

Reporting for specific materials, systems and methods

We require information from authors about some types of materials, experimental systems and methods used in many studies. Here, indicate whether each material, system or method listed is relevant to your study. If you are not sure if a list item applies to your research, read the appropriate section before selecting a response.

Materials & experimental systems

n/a	Involved in the study
☒	☐ Antibodies
☒	☐ Eukaryotic cell lines
☒	☐ Palaeontology and archaeology
☒	☐ Animals and other organisms
☒	☐ Clinical data
☒	☐ Dual use research of concern
☒	☐ Plants

Methods

n/a	Involved in the study
☒	☐ ChIP-seq
☒	☐ Flow cytometry
☒	☐ MRI-based neuroimaging
